# Pelvic neurofibroma in a patient presenting with pelvic pain and urinary frequency: A case report

**DOI:** 10.1016/j.radcr.2023.03.024

**Published:** 2023-04-19

**Authors:** Ali Hajihashemi, Mahsa Geravandi

**Affiliations:** Department of Radiology, Isfahan University of Medical Sciences, Daneshgah Avenue, Isfahan, Iran

**Keywords:** Neurofibroma, Pelvis, Nerve sheath tumor

## Abstract

Pelvic neurofibromas are benign and uncommon retroperitoneal masses. They arise from Schwann cells. One of the most common types of these benign tumors is intraneural neurofibromas, which are solitary, sporadic, and not associated with neurofibromatosis type 1. Here we discuss a case of pelvic neurofibroma in a 20-year-old male who presented with chronic pelvic pain. He had no positive family history of genetic disorders. On physical exam, just a partly firm mass without mobility in the hypogastric region was detected. Ultrasound and Computed tomography scan showed pelvic retroperitoneal mass superior to the urinary bladder with extension to the rectovesical pouch and invasion of the posterior wall and dome of the bladder. The patient underwent laparotomy revealing an infiltrative retroperitoneal mass with the invasion of the posterior wall, dome, and trigone of the bladder. Histopathological findings showed neurofibroma.

## Case report

A 20-year-old male presented to the general hospital and reported 6 months of progressively increasing pelvic pain, fatigue with occasional urinary frequency, and incontinency. He had no history of bowel or genital symptoms or complaint of limb numbness. There was no family history of genetic disorders.

In physical examination, the general appearance was normal. Heart rate and respiratory rate were 80 beats/min and 22 breaths/min respectively. The temperature was 37°c and the blood pressure was 145/100 mmHg.

No evidence of skin lesion was present. No neurological sign was detected. A partly firm mass without mobility in the hypogastric region was detected on abdominal palpation. The digital rectal examination did not show any abnormality.

At admission time, an examination of other systems showed no abnormalities.

Lab data revealed an erythrocyte sedimentation rate of 120 mm/h and C-reactive protein of 70 mg/L. The complete blood count test findings were normal. Blood urea nitrogen and serum creatinine were in the normal range.

Urinalysis showed microscopic hematuria (Blood 1+ and red blood cell 30-40/HPF).

On ultrasound, both kidneys showed normal appearance and size. No evidence of hydronephrosis was present. An infiltrative heterogeneous hypoechoic mass was seen over the urinary bladder dome with extension to the lateral wall of the pelvic cavity and rectovesical pouch, without a sharp margin measuring 125 × 45 × 65 mm. The mentioned mass had a pressure effect on the bladder wall, causing an undulating appearance of its wall. The prostate and seminal vesicles seemed to be normal. Soft tissue sarcoma, nerve sheath tumors, and retroperitoneal fibrosis were considered the differential diagnosis.

**Fig. 1:**
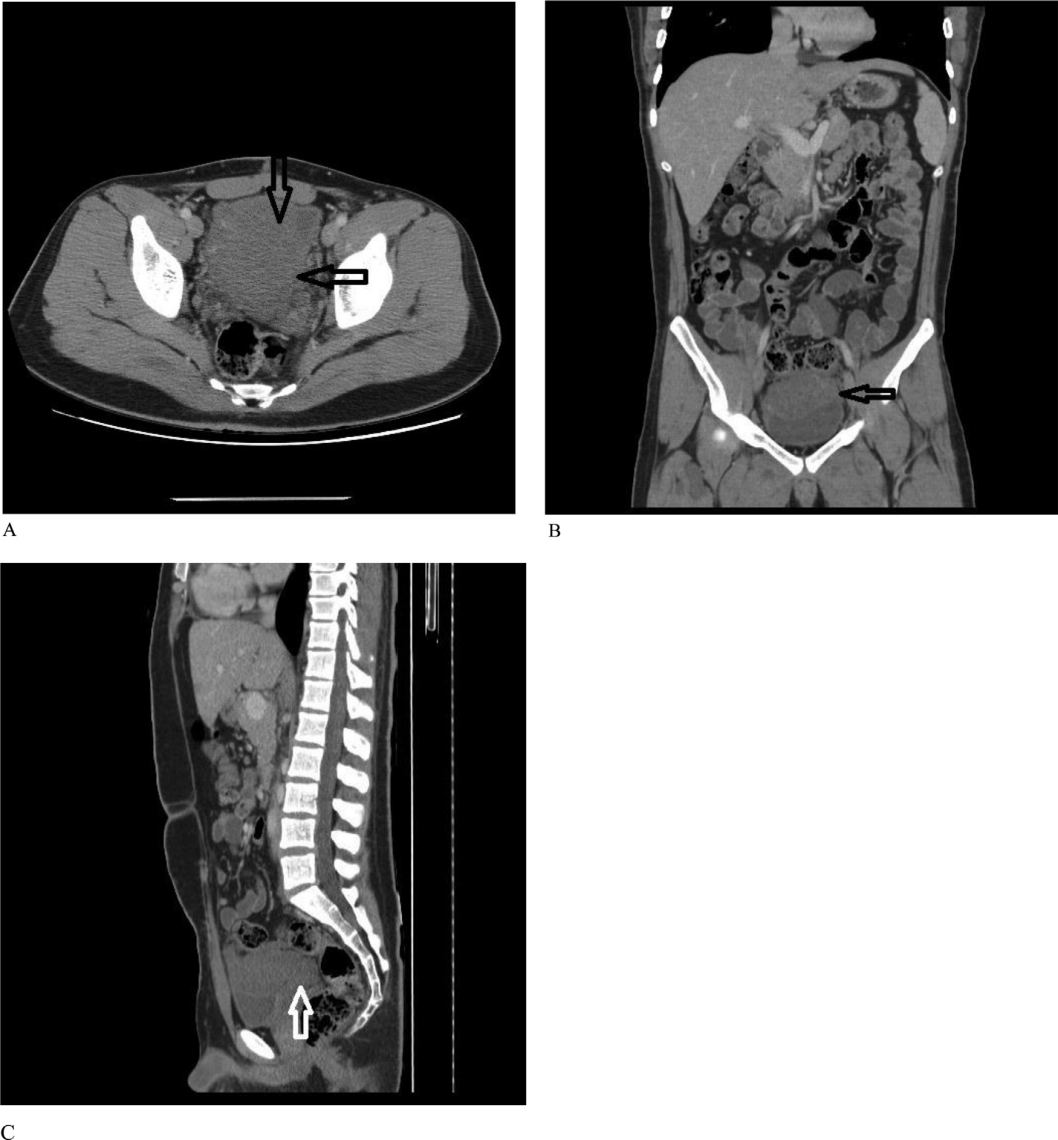
Axial (A), coronal (B), and sagittal (C) abdominopelvic CT scan with IV contrast: a large enhancing heterogeneous mass in the pelvic region between the urinary bladder and rectum (black arrows) with pressure effect and suspicious invasion to posterior and superior walls of the bladder (white arrow).

**Fig. 2:**
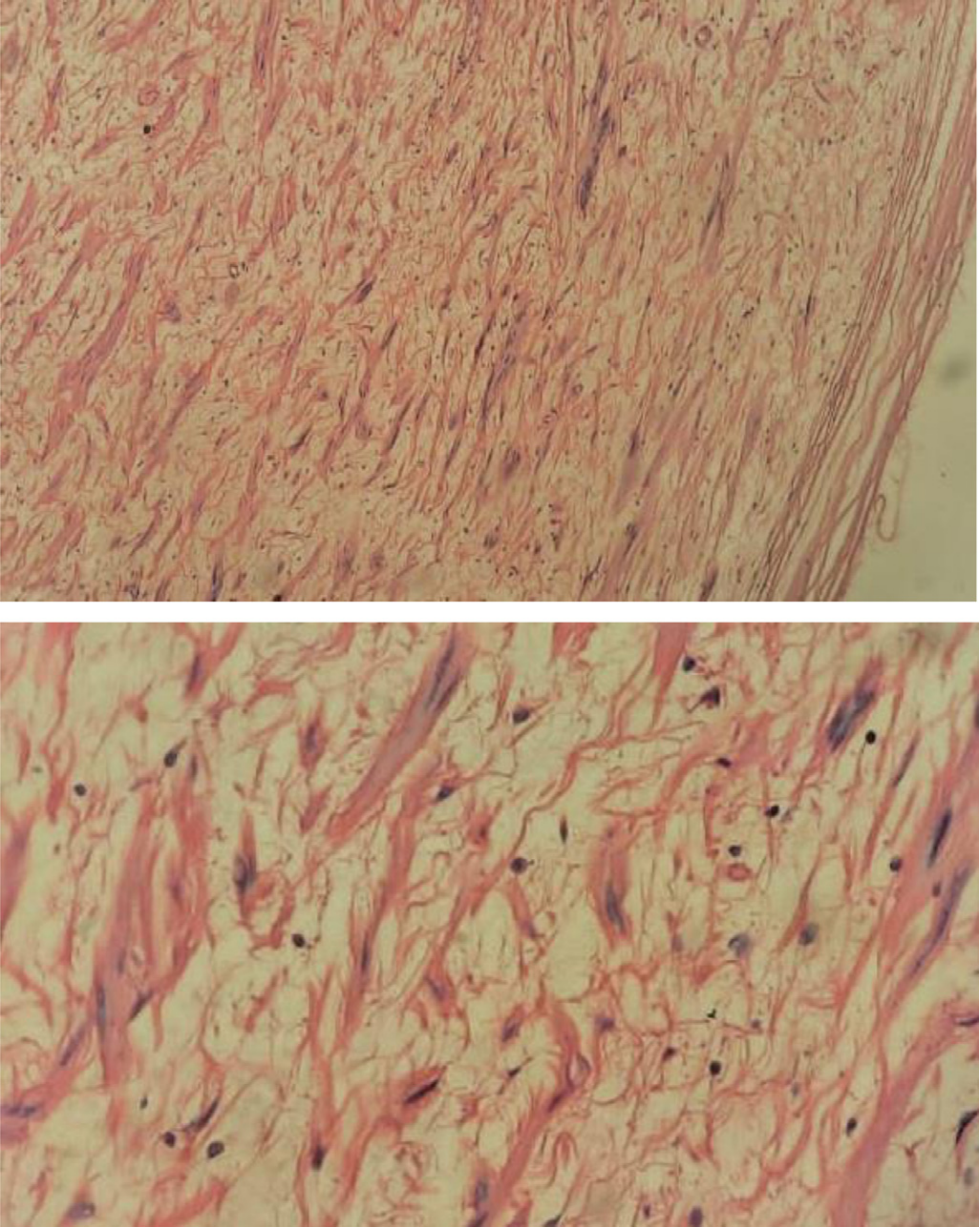
Histopathology with hematoxylin and eosin (H&E) staining of the lesion (A: 20× and B: 40×) of the lesion: the hypocellular proliferation of nerve elements including Schwann cells with interposed collagen bundles. These cells are spindle in shape with wavy nuclei and pointed ends.

A CT scan was recommended due to the patient's mass. It showed an enhanced soft tissue density mass in the pelvic cavity, superior to the urinary bladder with extension to the rectovesical pouch without calcification or macroscopic fat density. The mass invades the posterior wall and dome of the bladder. A few pre-aortic lymph nodes with a maximum short axis diameter of 13mm were also seen (Fig. 1). Tissue sampling was considered. Image-guided biopsy was done and a pathological examination revealed the diagnosis of neurofibroma (Fig. 2).

The patient underwent laparotomy with a midline incision. Intraoperative findings showed a large retroperitoneal pelvic mass which was infiltrative and had an invasion of the posterior wall, dome, and trigone of the bladder. In the following, excision of the bladder and seminal vesicles, until the prostate apex was done and sent specimens for frozen pathology which showed the Posterior urethra free of pathologic involvement. Radical cystectomy and orthotopic diversion were done. The patient's recovery was uneventful, and he was discharged 7 days after the drain was removed.

## Discussion

Neurofibromas are benign peripheral nerve sheath tumors that are usually solitary and sporadic. They arise from Schwann cells which are a type of glial cells surrounding neurons [1].

Neurofibromas subtypes include intraneural, cutaneous (localized & diffuse), plexiform, and massive diffuse soft tissue [2], [3], [4].

The most common type is intraneural neurofibromas, which are solitary and sporadic without association with neurofibromatosis type 1. However, the plexiform subtype of neurofibroma has a strong association with neurofibromatosis type 1. neurofibromas are found in various anatomical sites but rarely in the retroperitoneal location in the pelvis [3].

The peak incidence of localized intraneural neurofibromas (in sporadic form) is at 20-30 years old [5]. There is no difference in prevalence between men and women. In neurofibromatosis type 1, the manifestation is earlier than the sporadic form [6].

Clinical features depend on which anatomical area is involved in the retroperitoneum. Involving the urinary bladder can cause hematuria, dysuria, recurrent urinary tract infections, irritative symptoms, and pelvic mass; as in our case who presented with urinary frequency and hematuria [7].

Imaging modalities including ultrasonography, MRI, and computed tomography (CT) can be helpful in the early detection of these lesions, their extension, and in assessing possible malignant transformation [1,8].

On histology, exam neurofibroma is composed of collagenous matrix and spindle cells (medium-sized). The spindle cells are mostly positive for S-100 protein on immunostains as seen in our patient. Collagenous matrices are usually positive for Alcian blue. Neurofibromas are negative for cytokeratin and Epithelial membrane antigen [9].

Soft tissue sarcoma (low-grade rhabdomyosarcoma and leiomyosarcoma), leiomyoma, and malignant peripheral nerve sheath tumor (low grade) can mimic neurofibroma on histology. Clinical findings and histological and immunohistochemical exams are helpful for a definitive diagnosis [10].

The malignant transformation is rare in neurofibromas except for the plexiform subtype, so the prognosis of the majority of cases is very good. Local excision is the commonest treatment reported in the literature. The extent of excision depends on the tumor size and its invasion of adjacent structures [11].

## Availability of data and materials

Data sharing does not apply to this article as no datasets were generated or analyzed during the current study, but details from the clinical records are available from the corresponding author upon reasonable request.

## Ethics approval and consent to participate

Ethics approval and patient consent were obtained.

## Patient consent

Written informed consent for the publication of this case report was obtained from the patient.

## References

[1] Wong-You-Cheong JJ, Woodward PJ, Manning MA, Sesterhenn IA (2006). Neoplasms of the urinary bladder: radiologic-pathologic correlation. Radiographics.

[2] Hassell DS, Bancroft LW, Kransdorf MJ, Peterson JJ, Berquist TH, Murphey MD (2008). Imaging appearance of diffuse neurofibroma. Am J Roentgenol.

[3] Murphey MD, Smith WS, Smith SE, Kransdorf MJ, Temple HT. (1999). From the archives of the AFIP: imaging of musculoskeletal neurogenic tumors: radiologic-pathologic correlation. Radiographics.

[4] Cotran RS, Kumar V, Collins T (1999). Robbins Pathologic Basis of Disease.

[5] Pilavaki M, Chourmouzi D, Kiziridou A, Skordalaki A, Zarampoukas T, Drevelengas A. (2004). Imaging of peripheral nerve sheath tumors with pathologic correlation: pictorial review. Eur J Radiol.

[6] Knight SW, Knight TE, Santiago T, Murphy AJ, Abdelhafeez AH. (2022). Malignant peripheral nerve sheath tumors—a comprehensive review of pathophysiology, diagnosis, and multidisciplinary management. Children.

[7] Wang W, Montgomery E, Epstein JI. (2008). Benign nerve sheath tumors on urinary bladder biopsy. Am J Surg Pathol.

[8] Shonnard KM, Jelinek JS, Benedikt RA, Kransdorf MJ. (1992). CT and MR of neurofibromatosis of the bladder. J Comput Assist Tomogr.

[9] Bostwick DG, Cheng L. (2008).

[10] Cheng L, Scheithauer BW, Leibovich BC, Ramnani DM, Cheville JC, Bostwick DG. (1999). Neurofibroma of the urinary bladder. Cancer.

[11] Üre I, Gürocak S, Gönül II, Sözen S, Deniz N. (2013). Neurofibromatosis type 1 with bladder involvement. Case Rep Urol.

